# Acute idiopathic velopharyngeal insufficiency

**DOI:** 10.11604/pamj.2020.37.359.26072

**Published:** 2020-12-21

**Authors:** Malika El Omri, Oumaima Gabsi, Wassim Kermani, Mohamed Abdelkefi

**Affiliations:** 1 Ear, Nose and Throat Department and Cervical Surgery, Farhat Hached Hospital, Medicine University, Sousse, Tunisia

**Keywords:** Velopharyngeal, insufficiency, acute, idiopathic

## Abstract

Idiopathic velopharyngeal insufficiency is a rare clinical entity. Typical clinical features are rhinolalia and nasopharyngeal regurgitation. It is usually observed in children. We report a case of a 28-year-old female with this rare disorder. The symptoms were rhinolalia and regurgitation of liquids into the nose. The magnetic resonance imaging of the brain and upper cervical region was normal. The infectious serologies were negative.

## Introduction

The velopharynx is a complex structure that is limited by the soft palate and the lateral and posterior oropharyngeal walls. It is responsible for separation of the oropharynx and the nasopharynx, therefore playing an essential role in speech production and swallowing [1]. Velopharyngeal insufficiency (VPI) can lead to nasopharyngeal regurgitation and rhinolalia and can cause otologic diseases [2]. Congenital VPI is more frequent than acquired VPI, it´s often associated with other anomalies such as hereditary myopathy and cranio-facial malformations like hemifacial microsomia, down syndrome and palatine clefts. Acquired VPI can be seen after adenoidectomy in patients with palatine cleft, it can be also seen in some neurological disease such as ischemia, demyelinating diseases and pontocerebellar or brainstem expansive lesions (neoplastic or infectious), ischemia diseases [3]. It can also be associated with some neuromuscular diseases such as Moebius syndrome (which associates paresis of multiple cranial nerves) and dystrophinopathies (especially Duchenne´s disease) [2].

## Patient and observation

**Figure 1:**
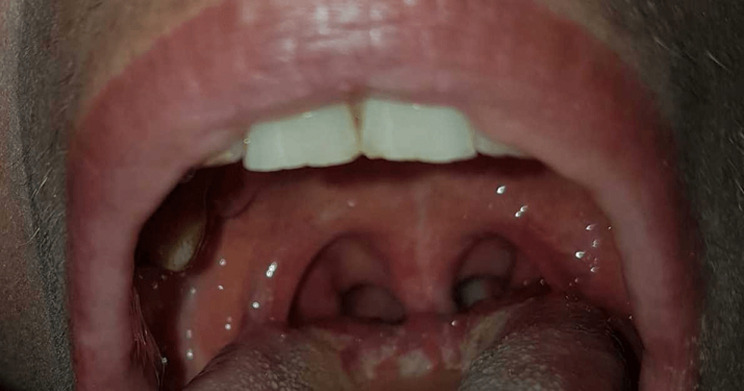
patient's palate photograph, obtained in phonation, shows bilateral velar paralysis

**Figure 2:**
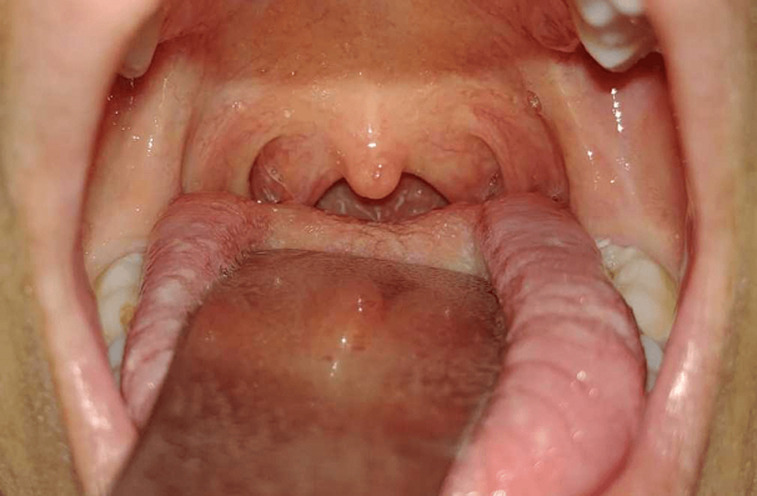
patient's palate photograph, obtained in phonation, shows symmetric velar contraction 3 weeks after symptoms´ onset

A 28-year-old woman presented with a 2-day history of sudden voice modification. She had no relevant medical past. She did not complain of nasal reflux. The rest of the systematic anamnesis was contributory for an episode of rhinitis that resolved spontaneously five days before symptoms´ onset. Physical examination revealed bilateral velar paralysis with open rhinolalia during phonation (Figure 1). No skin rash or other neurological focal signs were present. The gag reflex was preserved, and pharyngo-laryngeal and lingual mobility were normal. The exploration of the remaining cranial nerves was normal. Endoscopic examination of the pharynx revealed no masses and the rest of examination was normal. Magnetic resonance imaging (MRI) of the brain and upper cervical region demonstrated was normal, especially the intracranial portion of cranial nerves IX and X. Serologies were obtained for cytomegalovirus, herpes virus 1 and 2, varicella-zoster virus, hepatitis A virus, parvovirus B19 and Coxsackie A9i. They were negative. The patient followed a speech therapy. She didn’t receive any medical treatment. Full recovery was seen at 3 weeks follow-up (Figure 2).

## Discussion

VPI is usually observed in children; however, the cause of this disorder has not known. The etiology of the disorder is not known. The viral etiology seemed unlikely in the majority of cases [4]. Establishing idiopathic aetiology requires should exclude other conditions, mainly iatrogenic causes (including recent adenoidectomy) and pharmacological or toxic. Besides laboratory investigation, cerebral MRI must exclude demyelination and pontocerebellar or brainstem expansive lesions, ischemia [5]. The physiopathology of the condition remains to be elucidated. However, two mechanisms have been hypothesized: viral and vascular. First, the velopharyngeal muscles are innerved by the glossopharyngeal, the vagus and a branch of the facial nerve [6]. The vagus induces nasopharyngeal closure by palatal contraction and the other nerves transmit impulses to complete and harmonize the mobility of the soft palate [6, 7]. First, there are neurotropic viruses (Coxsackie, parvovirus B19, and paramyxovirus), and their neurological involvement could be explained by direct viral aggression. In the case of hepatitis A virus, which is known as non-neurotropic virus, an alternative mechanism to explain neurological involvement is wanted. The main hypothesis would be an immunologically mediated response [8].

Concerning measles, enterovirus and parvovirus B19, the formation of epitopes that induce mediate an autoimmune response after the reactivation of the latent virus (in the neural cells), has been proved experimentally. This could be a second potential mechanism that can explain neurological involvement [9]. Victorine tries to find a relation between acute VPI and *Borrelia burgdorferi*, one of the causative agents of Lyme disease or borreliosis. The diagnosis of neuroborreliosis could have been eventually confirmed by polymerase chain reaction identification of *Borrelia burgdorferi* in the cerebrospinal fluid and her patient´s history, presentation and ulterior evolution were rather inconsistent with this diagnosis [6]. The second hypothesis has been reported by Lapresle *et al*. There is may be an ischemia in the roots of the glossopharyngeal and vagus nerves. The schemia can be caused by many lesions such as vascular, tumoral, traumatic and inflammatory pathologies [10]. Thus, the examination of the velopharyngeal function is mandatory to suspect a high vagal or brainstem lesion when pharyngeal and palatal weakness are present in a patient [11]. We considered the paralysis of our patient as idiopathic because all the evaluations were negative. The prognosis in the situation is good with a high percentage of complete recovery, as in this case, and absence of recurrences. Further case reports would be useful to confirm the idiopathic and not malignant nature of this condition [4].

## Conclusion

Acute isolated VPI seems to be most often an idiopathic condition, but some cases could be associated with a pathologic process. Complementary imaging studies seem to be necessary and a close follow-up is imperative in order to eliminate progressive conditions of VPI.
